# Supplementary material for: Electrospinning vs. Electro-Assisted Solution Blow Spinning for Fabrication of Fibrous Scaffolds for Tissue Engineering
Source: Polymers (Basel). 2022 Dec 1;14(23):5254. doi: 10.3390/polym14235254 (PMC9740951; doi:10.3390/polym14235254)
Supplement: Supplementary file 1 [file polymers-14-05254-s001.zip › polymers-2035918-FIgure S1.pdf]

**Supplementary Materials**

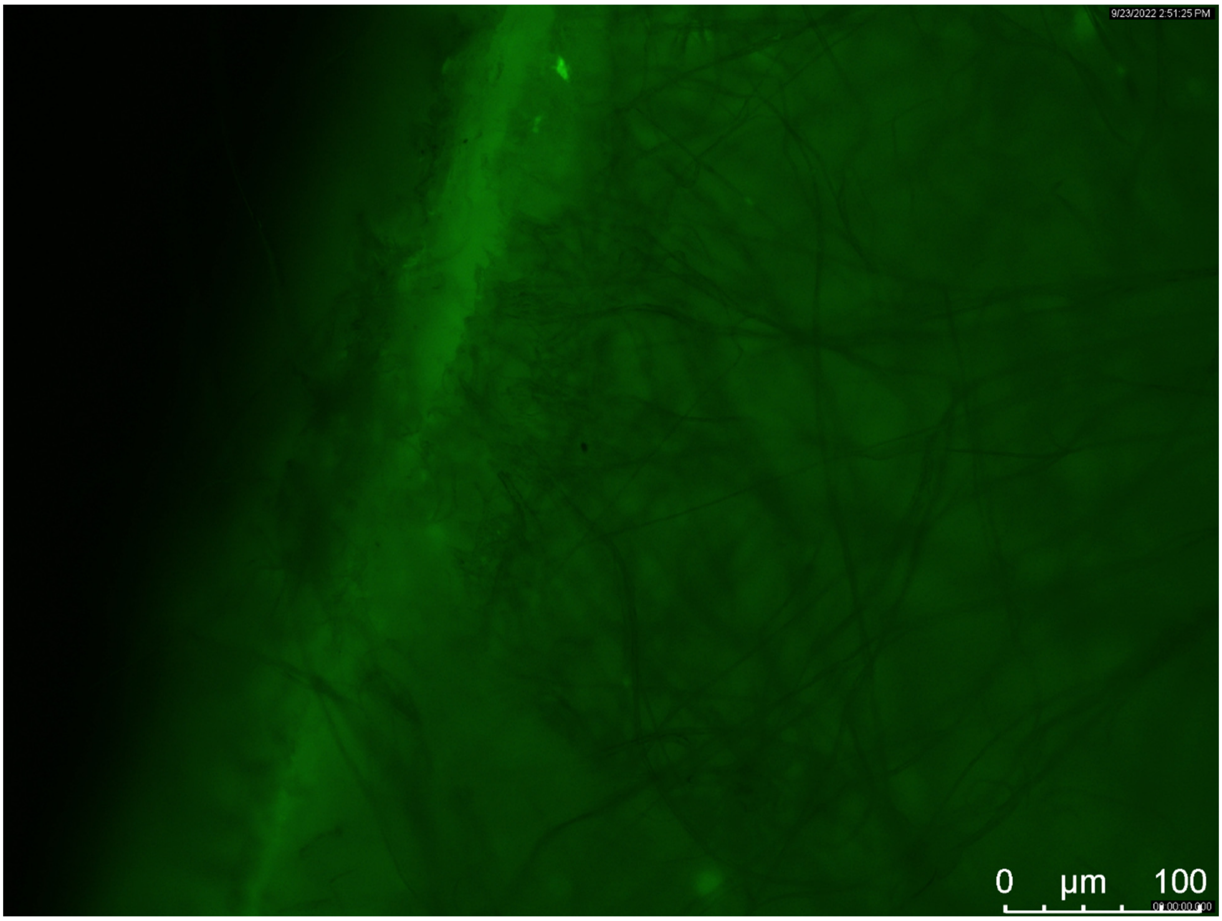


**Figure S1.** Fluorescent micrograph of EA-SBS-formed fibrous sample stained with fluorescein isothiocyanate (FITC).
